# Prevalence and risk factors of refractive error: a cross-sectional Study in Han and Yi adults in Yunnan, China

**DOI:** 10.1186/s12886-019-1042-0

**Published:** 2019-01-25

**Authors:** Meng Wang, Jiantao Cui, Guangliang Shan, Xia Peng, Li Pan, Zhimei Yan, Jie Zhang, Yong Zhong, Jin Ma

**Affiliations:** 1 Department of Ophthalmology, Peking Union Medical College Hospital, Chinese Academy of Medical Sciences & Peking Union Medical College, No. 1 Shuaifu Yuan, Dongcheng District, Beijing, 100730 China; 2 School of Medicine, Tsinghua University, Beijing, China; 3 Department of Epidemiology and Statistics, Institute of Basic Medical Sciences, Chinese Academy of Medical Sciences & Peking Union Medical College, Beijing, China; 4 Yunnan Center for Disease Control and Prevention, Kunming, Yunnan China; 5 Institute of Health Education of Kunming, Kunming, Yunnan China; 6 Center for Disease Control and Prevention of Wuhua District, Kunming, Yunnan China

**Keywords:** Refractive error, Han and Yi ethnicity, Prevalence, Risk factors

## Abstract

**Background:**

Few studies have investigated the prevalence of refractive error (RE) in older adults in China, and most have focused on East China. Our study determined the prevalence and risk factors of RE in Han and Yi adults aged 40–80 years in rural and urban areas in Yunnan Province, Southwest China.

**Methods:**

Our cross-sectional study is part of the China National Health Survey (CNHS). The age-adjusted prevalence rates of RE in Han and Yi adults aged 40–80 years in Yunnan were compared. We used a multivariate logistic regression model to identify risk factors for myopia and hyperopia.

**Results:**

Among 1626 participants, the age-adjusted prevalence rates of myopia, hyperopia, high myopia and astigmatism were 26.35% (95%CI 24.01–28.70%), 19.89% (95%CI 18.16–21.61%), 2.64% (95%CI 1.75–3.53%), and 56.82% (95%CI 54.31–59.34%). Compared to the Yi population, the Han population had higher prevalence of myopia (31.50% vs 16.80%, *p* < 0.0001), high myopia (3.34% vs 1.31%, *p* = 0.049) and astigmatism (60.07% vs 50.67%, *p* = 0.026) but lower prevalence of hyperopia (16.58% vs 27.37%, *p* < 0.0001). In the multivariate logistic regression, individuals aged 45–49 (*p* < 0.001), 50–54 (*p* < 0.001), 55–59 (*p* = 0.014), and 60–64 years (*p* = 0.005) had a lower myopia risk than those aged 40–44 years, and individuals aged 50–54 (*p* = 0.002), 55–59, 60–64 and 65 years and older (all *p* < 0.001) had a higher hyperopia risk than those aged 40–44 years. Myopia was also associated with height (*p* = 0.035), time spent in rural areas (*p* = 0.014), undergraduate/graduate education level (*p* = 0.001, compared with primary school or lower education level) and diabetes (*p* = 0.008). The Yi population had a higher risk of hyperopia than the Han population (*p* = 0.025). Moreover, hyperopia was related to time spent in rural areas (*p* < 0.001) and pterygium (*p* = 0.019).

**Conclusions:**

Our study investigated the overall prevalence of RE in older adults in rural and urban areas of Southwest China. Compared to the Yi population, the Han population had a higher prevalence of myopia, high myopia and astigmatism but a lower risk of hyperopia. The prevalence of myopia in the Han population in underdeveloped Southwest China was similar to that of residents in East China or of Chinese Singaporeans under urban or rural settings.

## Background

Refractive error (RE) is one of the most important ocular anomalies. Uncorrected RE is the leading cause of visual impairment [1]. In recent decades, myopia has become highly epidemic in children and young adults in East Asia. A Korean study reported a myopia prevalence of 83.3% in young adults aged 19 years [2]. In China, the myopia prevalence was 73.1% in teenagers 15 years of age in Guangzhou [3] and 95.5% among undergraduate or graduate students in Shanghai [4]. For older adults, studies in Singapore (Tanjong Pagar Study: myopia 38.7%), [5] Japan (Tajimi Study: myopia 41.8%), [6] India (Andhra Pradesh Eye Disease Study: myopia 34.6%), [7] and Burma (Meiktila Eye Study: myopia 42.7%) [8] showed a relatively higher prevalence of myopia than that in the United States (Multiethnic Study of Atherosclerosis: myopia 25.1%) [9] and Australia (Victoria Visual Impairment Project: myopia 17.0%) [10]. Studies in multiethnic countries revealed that Chinese individuals have a higher myopia prevalence and a lower hyperopia prevalence. In the United States, the prevalence of myopia in Chinese subjects is three times that in Hispanic subjects [9]. In Singapore, Chinese people have a higher risk of myopia and a lower risk of hyperopia than Malay and Indian people [11]. However, there are few studies investigating the RE prevalence in older adults in mainland China, and most of these studies were conducted in Eastern China with a large variation between the reported prevalence rates [5, 12–17].

The Chinese population is composed of the Han ethnicity (91.51%) and 55 ethnic minorities [18]. Previous studies indicated differences in the prevalence of myopia between the Han population and the ethnic minorities. An epidemiologic study in Xinjiang found that the prevalence of myopia is Han > Hui > Uygur in children aged 4–19 years [19]. Another study, involving undergraduates in Shanghai, reported a higher risk for myopia in Han individuals than in subjects from ethnic minorities [4]. Apart from Yunnan Minority Eye study, [17] few studies have investigated the difference in the prevalence of myopia between the Han population and the ethnic minorities in elder adults. Located on the southwestern border of China, Yunnan is crossed by the Tropic of Cancer. Yi is the seventh largest ethnicity of China, and 57.85% of the Yi population live in Yunnan Province as the largest ethnic minority in Yunnan [20]. Previous studies revealed the significant difference between the Yi and Han populations in health-related quality of life, [21] metabolic syndrome, [22] blood cell parameters [23] and certain gene loci [24].

The Yunnan Minority Eye Study has investigated the prevalence of myopia in rural areas in Yunnan [17]. To obtain a more comprehensive understanding of refractive error in Southwest China, our cross-sectional study, as part of the China National Health Survey (CNHS), described the overall prevalence of myopia, hyperopia, high myopia and astigmatism with sampling in a large city, a county site and a rural section containing Han and Yi populations aged 40–80 years in Yunnan. Furthermore, we explored the risk factors for myopia and hyperopia.

## Methods

### Study population

The CNHS was conducted by the Chinese Academy of Medical Sciences to evaluate the Physiological Constant and Health Condition in Chinese. The part reported in this study was conducted in Yunnan in 2015. The CNHS used a multistage cluster sampling method. According to the level of urbanization, three locations were randomly chosen for sampling in this cross-sectional study, including a large city (Health Examination Center of Wuhua District in Kunming City), a county seat (Xiuping Community Hospital in Luquan County), and a rural section (Chepanying Town in Luquan County). In each selected area, different districts were selected as sampling units.

### Data collection

#### Questionnaire and routine physical examination

A questionnaire about demographic information and health history was administered during a comprehensive interview by well-trained interviewers. The questionnaire included information about age, sex, ethnicity, birthplace, current residence, migration date, education level, occupation, income per month, smoking and drinking practices, occupational and leisure-time physical activities, and medical history (including hypertension and diabetes status). An assessment of height, weight, blood pressure, and fasting blood glucose was included in routine physical examinations. Height was measured to the nearest 0.1 cm using a fixed stadiometer and in the standing position by bioelectrical impedance analysis (BIA) with a commercially available body composition analyser (BC-420, TANITA, Japan) with participants in lightweight clothes. A digital automatic blood pressure monitor was used to measure systolic and diastolic blood pressure, and the average of three measurements was recorded. Blood samples were drawn after fasting overnight for at least 8 h and were then immediately processed, refrigerated, transferred and assessed in the laboratory at the General Hospital of Chinese Peoples’ Liberation Army, Beijing. Body mass index (BMI) was calculated using the formula weight (kg)/height (m)^2^.

#### Eye examination

Well-trained ophthalmologists performed eye examinations on our participants. A logarithm of the minimum angle of resolution E chart (Wehen Co., Ltd., Guangzhou, China) was used for visual acuity measurement at 4 m. An auto ref-keratometer (ARK-510A, Nidek Co., Ltd., Tokyo, Japan) was used to measure noncycloplegic refraction and corneal curvature radium. The anterior segment of the eye was examined with a portable hand-held slit-lamp (KJ5S2, Suzhou Kangjie Medical Co. Ltd., Jiangsu, China).

#### Inclusion and exclusion criteria

Only participants whose parents were both Han or both Yi were included in our study. The proportions of Han and Yi participants in our study were similar to the natural proportions of the population distribution at the survey sites. Only people who had lived in their current residence for more than 1 year were included. Psychiatric patients, pregnant women and active duty soldiers were excluded. Thus, 1860 participants aged 40–80 years old were eligible for our study; 1665 participants completed the questionnaire and eye examination, with a response rate of 89.52%. After excluding individuals with a cataract or myopic surgical history, data of 1626 participants were included in the final analysis.

#### Stratification standard

All participants were divided into six age groups, which were 40–44, 45–49, 50–54, 55–59, 60–64, and 65+ years old. Education level was divided into three groups including primary school and lower, middle/high school, and undergraduate/graduate. Occupation information was divided into close-work (including workmen and government workers), and non-close-work (including farmers, waiters and domestic workers). Additionally, participants were divided into never-smokers and ever-smokers (including current smokers and former smokers). Alcohol consumption was divided into two categories: never-drinkers and ever-drinkers (including current drinkers and former drinkers). We merged occupational and leisure-time physical activity into an activity level and regrouped it into three levels: low, moderate and high.

#### Definitions of RE

In our study, myopia was defined as spherical equivalent (SE) < − 0.5D, and hyperopia was defined as SE > 0.5D. High myopia was defined as SE < − 6.0D. Astigmatism was defined as ≥0.5D of the cylinder.

#### Statistics

A high correlation between right and left eyes was found in our study (Spearman correlation test, *p* < 0.0001, r = 0.8291). We obtained similar statistical analysis results between right and left eyes and only reported the results of the right eye for concision. The Chi-square test and Mann-Whitney test were used to compare the demographic differences between Han and Yi populations. A linear regression model was used to compare the SE and RE prevalence among different age groups. The difference in RE distributions between ethnicities among different age groups was tested with a Chi-square test. The Wilcoxon signed rank test was used to test the difference in SE among different age groups between ethnicities. The risk factors for RE were identified through a multivariate logistic regression analysis. Myopic and hyperopic individuals were compared to emmetropic individuals. The age-standardized prevalence was based on the Sixth National Population Census of the People’s Republic of China. A *p* value less than 0.05 was considered significant. Statistical analyses were performed using Stata version 13.1 (StataCorp, USA) and Statistical Analysis System (SAS) version 9.4; figures were created using GraphPad Prism 5.0 (Graphpad Software Inc., USA).

## Results

### Characteristics of Han and Yi adults

In total, 1626 participants aged 40–80 years old, comprising 1085 Han participants and 541 Yi participants, were included in the final analysis (Table 1). The sex and age compositions were similar between the Han and Yi populations (*p* = 0.881 and *p* = 0.081 respectively). Additionally, no difference was found in lifestyles, including smoking and drinking practices, between the Han and Yi populations (*p* = 0.112 and *p* = 0.130 respectively). However, the Han and Yi participants differed in many demographic aspects in our study. The Han population was taller and heavier and had a higher BMI than the Yi population (all *p* < 0.001). A greater proportion of the Han population was born and now lived in an urban area and had a shorter time spent in rural areas (all *p* < 0.001). Moreover, more Han participants were influenced by hypertension (40.01% vs 34.01%, *p* = 0.006) and diabetes (5.99% vs 2.77%, *p* = 0.005). There were slight differences between the Han and Yi populations with respect to activity levels, and a higher proportion of the Yi population reported moderate and heavy activity levels (light: 15.21% vs 10.72%; moderate: 77.05% vs 80.59%; heavy: 7.74% vs 8.69%; *p* = 0.043).

**Table 1:** Characteristics of the 1626 participants

	Han *n* = 1085	%	Yi *n* = 541	%	*P* value
Sex					0.881
Male	325	29.95	164	30.31	
Female	760	70.05	377	69.69	
Age (y)					0.081
40–44	128	11.80	78	14.42	
45–49	204	18.80	126	23.29	
50–54	212	19.54	104	19.22	
55–59	170	15.67	66	12.20	
60–65	178	16.41	82	15.16	
65+	193	17.79	85	15.71	
Height (cm)	158.34 + 7.52		156.89 + 7.11		0.0006
Weight (kg)	60.68 + 9.61		57.41 + 10.15		< 0.0001
BMI (kg/m^2^)	24.15 + 3.01		23.26 + 3.38		< 0.0001
Current Residence					< 0.0001
Urban	778	71.71	191	35.30	
Rural	307	28.29	350	64.70	
Birth Place					< 0.0001
Urban	322	29.68	4	0.74	
Rural	763	70.32	537	99.26	
Time spent in rural areas^1^	30.00 + 24.20		51.23 + 12.59		< 0.0001
Education					< 0.0001
Primary school or lower	446	41.11	409	75.60	
Middle/high school	497	45.81	118	21.81	
Undergraduate/graduate	142	13.09	14	2.59	
Occupation					< 0.0001
Non-close	645	59.45	485	89.65	
Close	440	40.55	56	10.35	
Income^2^ (¥)					< 0.0001
< 800/month	316	29.40	386	71.48	
800–2000/month	372	34.60	101	18.70	
> 2000/month	387	36.00	53	9.81	
Hypertension	445	40.01	184	34.01	0.006
Diabetes	65	5.99	15	2.77	0.005
Smoking					0.112
Never	837	77.14	436	80.59	
Past/current	248	22.86	105	19.41	
Drinking					0.130
Never	762	70.23	360	66.54	
Past/current	323	29.77	181	33.46	
Activity level					0.043
Light	165	15.21	58	10.72	
Moderate	836	77.05	436	80.59	
Heavy	84	7.74	47	8.69	

Note: 1) Twenty-five participants had missing data regarding time spent in rural areas; 2) 11 participants had missing income data. The Chi-square test and Mann-Whitney test were used to test the demographic differences between the Han and Yi populations

### Prevalence of RE in the Han and Yi populations

The age-adjusted prevalence rates of myopia, hyperopia, high myopia and astigmatism were 26.35% (95%CI 24.01–28.70%), 19.89% (95%CI 18.16–21.61%), 2.64% (95%CI 1.75–3.53%), and 56.82% (95%CI 54.31–59.34%) (Table 2). Moreover, there were differences in the prevalence of different RE values between the Han and Yi populations. The age-adjusted prevalences of myopia (31.50% vs 16.80%, *p* < 0.0001), high myopia (3.34% vs 1.31%, *p* = 0.049) and astigmatism (60.07% vs 50.67%, *p* = 0.026) were higher in the Han population than in the Yi population. However, the age-adjusted prevalence of hyperopia in the Yi population was higher than that in the Han population (27.37% vs 16.58%, *p* < 0.0001).

**Table 2:** Crude and age-adjusted prevalence of different REs

*N* = 1626	n	Crude Rate	Age-adjusted Rate	95%CI lower	95%CI upper	*P* value
Myopia						
Total	379	23.31%	26.35%	24.01%	28.70%	
Han	302	27.83%	31.50%	27.56%	35.45%	< 0.0001
Yi	77	14.23%	16.80%	12.81%	20.78%	
Hyperopia						
Total	375	23.06%	19.89%	18.16%	21.61%	
Han	216	19.91%	16.58%	14.29%	18.87%	< 0.0001
Yi	159	29.39%	27.37%	22.97%	31.78%	
High myopia						
Total	39	2.40%	2.64%	1.75%	3.53%	
Han	33	3.04%	3.34%	2.08%	4.60%	0.049
Yi	6	1.11%	1.31%	0.19%	2.43%	
Astigmatism						
Total	939	57.75%	56.82%	54.31%	59.34%	
Han	667	61.47%	60.07%	55.11%	65.02%	0.026
Yi	272	50.28%	50.67%	44.32%	57.02%	

### Distribution of RE and SE in different age groups

**Fig. 1:**
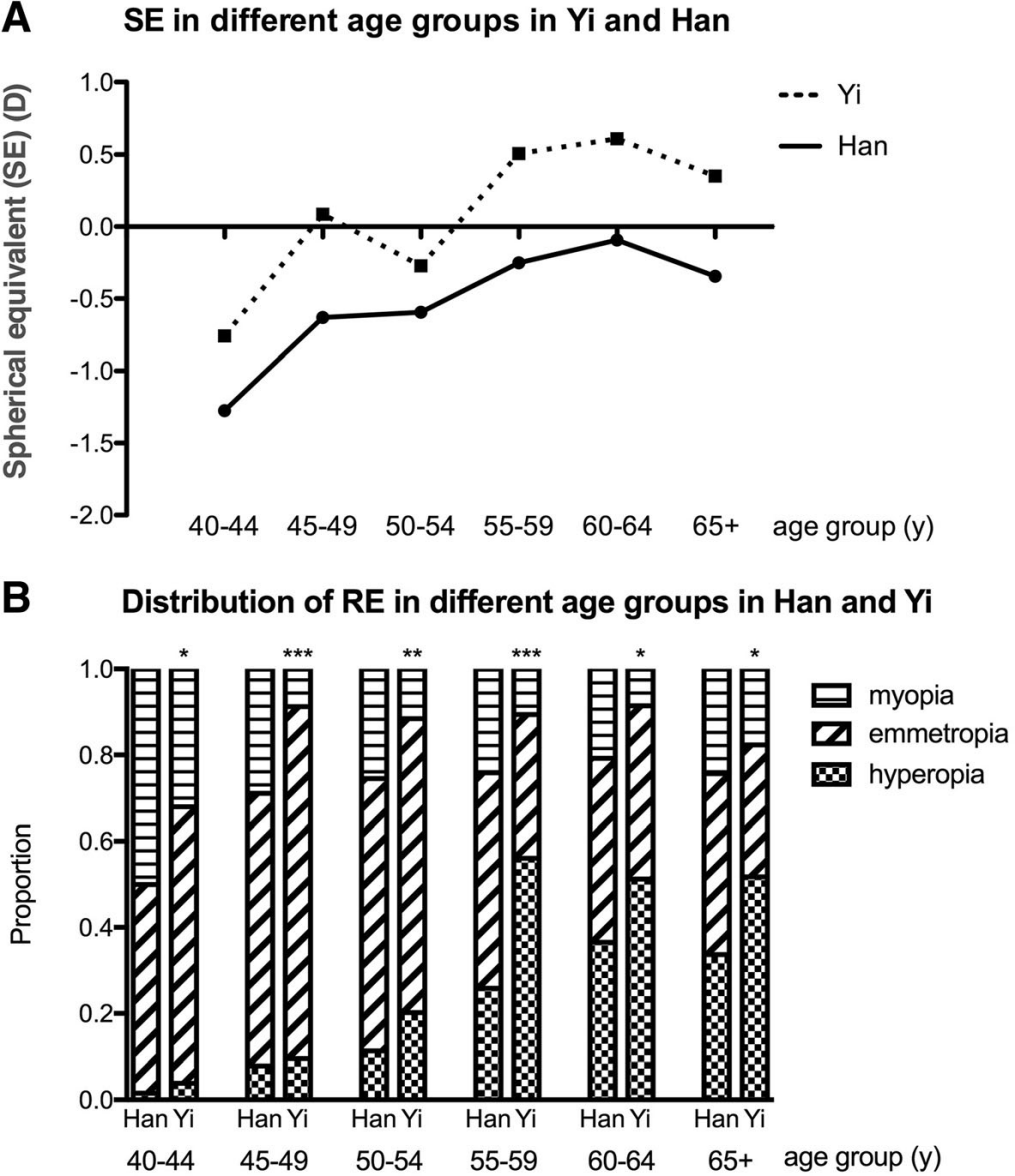
Spherical equivalent (SE) and distribution of refractive error (RE) in different age groups in Yi and Han populations. **a**: SE in different age groups in Han and Yi populations; **b**: Distribution of RE in different age groups in Han and Yi populations. Chi-square tests were used to test the distribution difference in the prevalence of RE between Han and Yi populations (*p* < 0.05 *, *p* < 0.01 **, *p* < 0.001 ***)

Participants included in the final analysis were divided into six age groups, which were 40–44, 45–49, 50–54, 55–59, 60–64, and 65 and over years old. The mean SE increased in the Han population as the age increased (*p* = 0.034), and the Yi population showed an increasing trend with age, although without statistical significance (*p* = 0.052) (Fig. 1a). The Yi population had the highest SE level in all age groups compared to the SE levels of the Han population (*p* = 0.031). The prevalence of hyperopia increased with ageing in both the Han (*p* = 0.002) and Yi populations (*p* = 0.012). The distributions of RE differed between the Han and Yi ethnicities for all age groups (40–44 *p* = 0.033, 45–49 *p* < 0.0001, 50–54 *p* = 0.005, 55–59 p < 0.0001, 60–64 *p* = 0.018, 65+ *p* = 0.017) (Fig. 1b).

### Risk factors for myopia and hyperopia

A multivariate logistic regression with 12 indicators including ethnicity, sex, age period, height, length of time spent in rural areas, education level, activity level, occupation, income, pterygium status of the same eye, diabetes status and smoking practice was used to assess risk factors for myopia and hyperopia. Myopia and hyperopia results were compared to those of emmetropic individuals, and the results are presented in Table 3. Ethnicity was not a risk factor for myopia (OR 0.77, 95%CI 0.55–1.08, *p* = 0.127) but was associated with hyperopia, and Yi ethnicity (compared to Han ethnicity) was a risk factor for hyperopia (OR 1.45, 95%CI 1.05–2.00, *p* = 0.025).

**Table 3:** Multivariate regression results for the right eye in all participants

	Myopia				Hyperopia			
	Odds Ratio	95%CI lower	95%CI upper	*P* value	Odds Ratio	95%CI lower	95%CI upper	*P* value
Ethnicity	0.77	0.55	1.08	0.127	1.45	1.05	2.00	0.025
Sex	1.00	0.58	1.73	0.994	1.84	0.99	3.40	0.052
Age range								
40–44	1.00				1.00			
45–49	0.44	0.29	0.67	0.000	2.31	0.86	6.20	0.096
50–54	0.43	0.28	0.66	0.000	4.54	1.73	11.90	0.002
55–59	0.55	0.34	0.89	0.014	14.85	5.69	38.79	0.000
60–64	0.49	0.30	0.81	0.005	18.59	7.16	48.25	0.000
65+	0.76	0.47	1.22	0.255	21.18	8.07	55.56	0.000
Height	0.97	0.95	1.00	0.035	0.98	0.95	1.00	0.073
Time spent in rural areas	0.99	0.98	1.00	0.014	1.02	1.01	1.03	0.000
Education								
< =Primary school	1.00				1.00			
Middle/high school	1.08	0.77	1.51	0.667	0.83	0.58	1.19	0.311
Undergraduate/graduate	2.50	1.46	4.25	0.001	0.97	0.46	2.05	0.942
Activity level	0.92	0.69	1.22	0.554	0.98	0.72	1.35	0.923
Occupation	1.33	0.94	1.90	0.110	1.28	0.81	2.03	0.285
Income	1.11	0.89	1.40	0.351	1.26	0.98	1.62	0.074
Pterygium	1.00	0.53	1.89	0.992	1.80	1.10	2.93	0.019
Diabetes	2.11	1.21	3.66	0.008	0.83	0.43	1.64	0.600
Smoking	1.27	0.76	2.13	0.361	1.68	0.94	2.98	0.078

Note: A multivariate logistic regression model with 12 indicators (including ethnicity, sex, age range, height, length of time spent in rural areas, education level, activity level, occupation, income, pterygium status of the same eye, diabetes status and smoking practice) was used to assess risk factors for myopia and hyperopia. Myopic and hyperopic individuals were compared to emmetropic individuals. The OR of ethnicity was Yi/Han. The OR of sex was female/male. The OR of occupation was close-workers/non-close-workers. The OR of pterygium was the eye with pterygium/the eye without pterygium. The OR of diabetes was individuals with diabetes/individuals without diabetes. The OR of smoking was individuals who had smoked/individuals who had never smoked

**Fig. 2:**
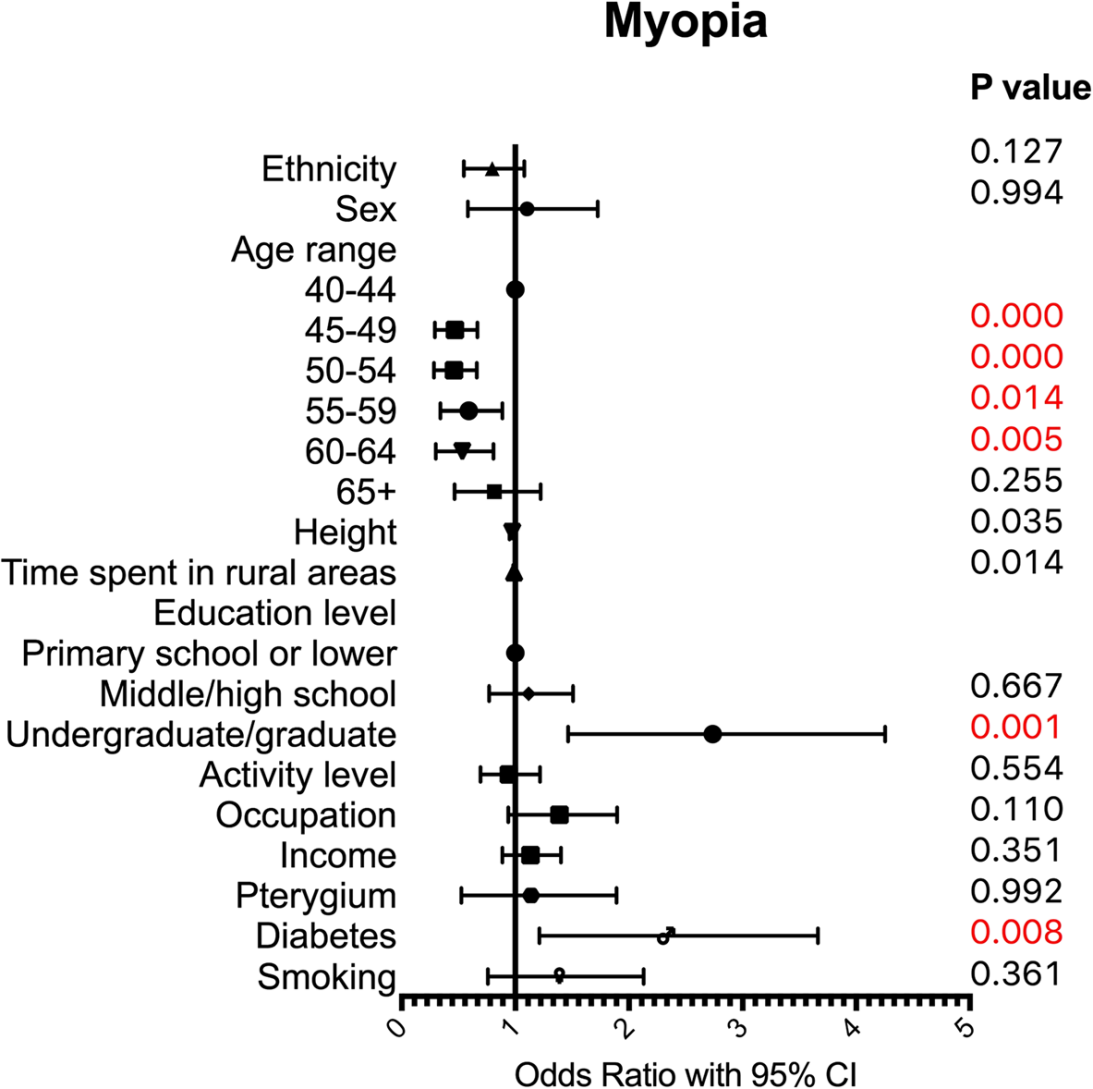
Multivariate logistic regression results for myopia. A multivariate logistic regression model with 12 indicators (including ethnicity, sex, age range, height, length of time spent in rural areas, education level, activity level, occupation, income, pterygium status of the same eye, diabetes status and smoking practice) was used to assess risk factors for myopia. Myopic individuals were compared to emmetropic individuals. The OR of ethnicity was Yi/Han. The OR of sex was female/male. The OR of occupation was close-workers/non-close-workers. The OR of pterygium was the eye with pterygium/the eye without pterygium. The OR of diabetes was individuals with diabetes/individuals without diabetes. The OR of smoking was individuals who had smoked/individuals who had never smoked.

For myopia (Fig. 2), compared to participants aged 40–44 years, those aged 45–49 (OR 0.44, 95%CI 0.29–0.67, *p* < 0.0001), 50–54 (OR 0.43, 95%CI 0.28–0.66, *p* < 0.0001), 55–59 (OR 0.55, 95%CI 0.34–0.89, *p* = 0.014), and 60–64 years (OR 0.49, 95%CI 0.30–0.81, *p* = 0.005) had a lower risk of myopia. Compared to those with a primary school or lower education level, participants who had an undergraduate/graduate education level had a much higher risk of myopia (OR 2.50, 95%CI 1.46–4.25, *p* = 0.001). Myopia was also identified to be associated with height (OR 0.97, 95%CI 0.95–1.00, *p* = 0.035) and time spent in rural areas (OR 0.99, 95%CI 0.98–1.00, *p* = 0.014). Moreover, individuals with diabetes had a much higher risk of myopia than those without diabetes (OR 2.11, 95%CI 1.21–3.66, *p* = 0.008).

**Fig. 3:**
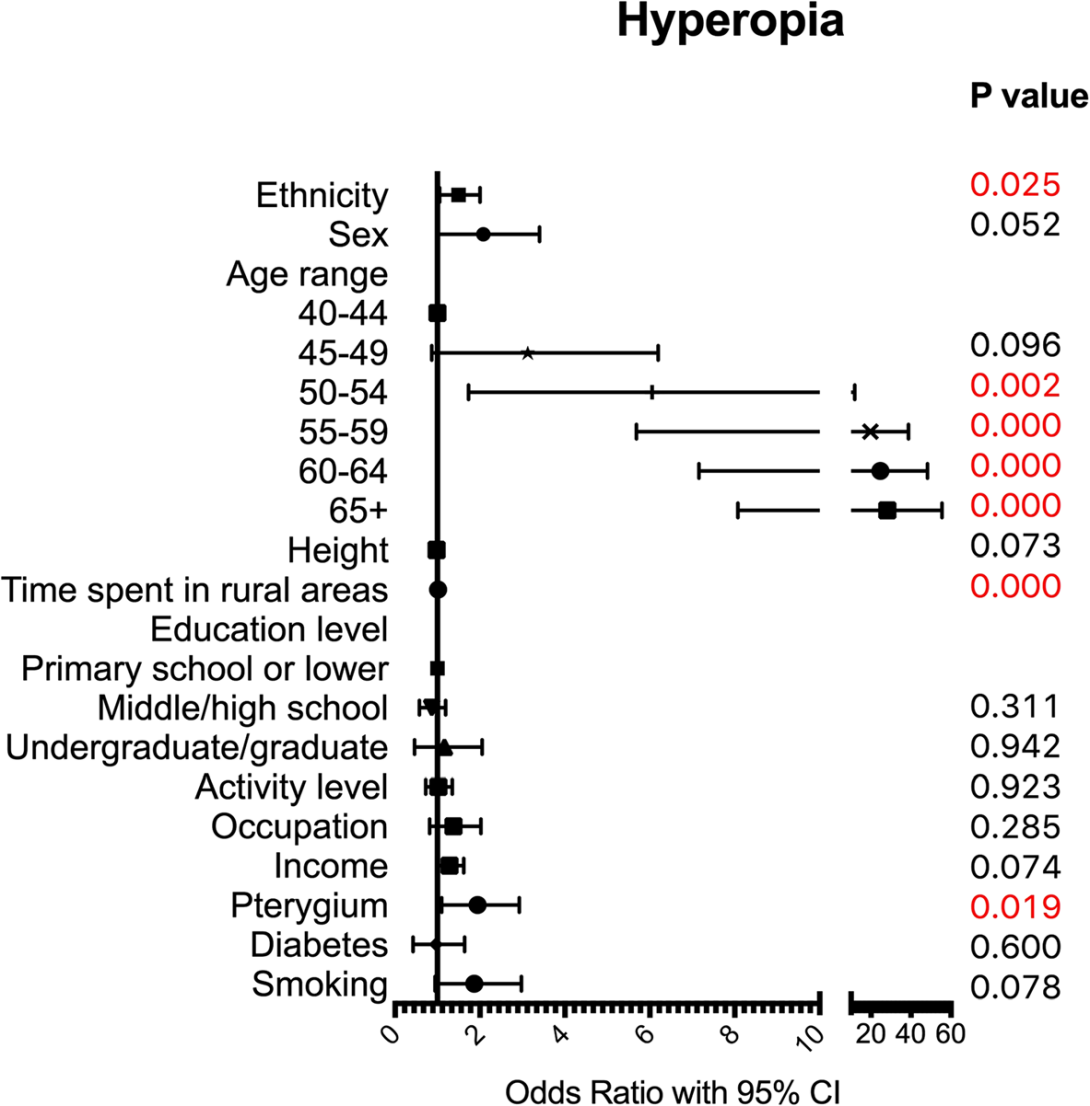
Multivariate logistic regression results for hyperopia. A multivariate logistic regression model with 12 indicators (including ethnicity, sex, age range, height, length of time spent in rural areas, education level, activity level, occupation, income, pterygium status of the same eye, diabetes status and smoking practice) was used to assess risk factors for hyperopia. Hyperopic individuals were compared to emmetropic individuals. The OR of ethnicity was Yi/Han. The OR of sex was female/male. The OR of occupation was close-workers/non-close-workers. The OR of pterygium was the eye with pterygium/the eye without pterygium. The OR of diabetes was individuals with diabetes/individuals without diabetes. The OR of smoking was individuals who had smoked/individuals who had never smoked.

For hyperopia (Fig. 3), apart from ethnicity, age was also significantly related to the occurrence of hyperopia. Compared to those aged 40–44 years, in individuals aged 45–49 years, the risk of hyperopia did not increase. However, individuals aged 50–54 (*p* = 0.002), 55–59, 60–64, and 65+ years (all *p* < 0.0001) had a much higher risk of hyperopia than those aged 40–44 years, with an increasing OR with ageing; furthermore, the OR for the 65+ age group reached 21.28 (95%CI 8.07–55.56, *p* < 0.0001). Time spent in rural areas also changed the risk of hyperopia slightly (OR 1.02, 95%CI 1.01–1.03, *p* < 0.0001). Moreover, the pterygium status of the same eye increased the hyperopia risk, with an OR equal to 1.80 (95%CI 1.10–2.93, *p* = 0.019).

## Discussion

Our study described and compared the prevalence of RE in Han and Yi populations aged 40–80 years in Yunnan. The overall myopia prevalence among the subjects in our study was 26.35% (95%CI 24.01–28.70%), which is even higher than the prevalence of myopia in older adults in Beijing, [13] Suzhou [15] and Shanghai, [12] but lower than that in Chinese individuals in Singapore [5] and Guangzhou (Table 4) [14].

**Table 4:** Comparison of the reported prevalence of RE in selected population-based studies in elder studies

Studies	n	Population	Age (y)	Myopia (%)	Myopia SE	High myopia (%)	High myopia SE	Hyperopia (%)	Hyperopia SE
Handan Eye Study [16]	6491	Chinese (Most Han)	40–79	18.8	<−0.5D	1.5	<−5.0D	23.5	> 0.5D
Yunnan Minority Eye Study [17]	2205	Chinese (Han ethnicity)	≥50	10.3	<−0.5D	2.4	<−6.0D	NR	NR
Yunnan Minority Eye Study [17]	2208	Chinese (Yi ethnicity)	≥50	8.1	<− 0.5D	1.6	<−6.0D	NR	NR
Beijing Eye Study [13]	4319	Chinese	40–90	22.9	<− 0.5D	2.6	<− 6.0D	20.0	> 0.5D
Liwan Eye Study [14]	1269	Chinese	≥50	32.3	<− 0.5D	5.0	<− 5.0D	40.0	> 0.5D
Suzhou Eye Study [15]	4795	Chinese	≥60	21.1	<− 0.5D	2.5	<− 5.0D	NR	NR
Shanghai Eye Study [12]	6099	Chinese	≥50	22.8	<−0.5D	4.6	<− 6.0D	48.5	> 0.5D
Tanjong Pagar Study [5]	1232	Chinese (Singapore)	40–79	38.7	<−0.5D	9.1	<−5.0D	28.4	> 0.5D
Singapore Malay Eye Survey [28]	2974	Malay (Singapore)	40–79	26.2	<−0.5D	3.9	<−5.0D	27.4	> 0.5D
Singapore Indian Eye Study [27]	2805	Indian (Singapore)	40–79	28.0	<−0.5D	4.1	<−5.0D	35.9	> 0.5D
Meiktila Eye Study [8]	1863	Burmese	≥40	42.7	<−1.0D	6.5	<−6.0D	15.0	> 1.0D
Tajimi Study [6]	3021	Japanese	≥40	41.8	<−0.5D	8.2	<−5.0D	27.9	> 0.5D
Six Villages in Sumatra [31]	358	Indonesian	≥40	34.1	≤ − 0.5D	NR	NR	32.1	≥0.5D
Andhra Pradesh Eye Disease Study [7]	3642	Indian	40–92	34.6	<−0.5D	4.5%	<−5.0D	18.4	> 0.5D
Shahroud Eye Cohort Study [29]	4864	Iranian	40–64	30.2	≤ − 0.5D	1.9	<−6.0D	35.6	> 0.5D
Mongolian Eye Study [30]	1617	Mongolian	≥40	17.2	<−0.5D	NR	NR	49.9	> 0.5D
National Health and Nutrition Examination Survey 1999–2004 [58]	7357	American	≥40	31.0	≤ − 1.0D	6.0	≤ − 5.0D	5.3	≥3.0D
Multiethnic Study of Atherosclerosis [9]	4430	American	45–84	25.1	≤ − 1.0D	4.6	≤ − 5.0D	38.2	≥1.0D
Barbados Eye Study [26]	4709	Barbados-born Black adults	40–84	21.9	<−0.5D	NR	NR	46.9	> 0.5D
Victoria Visual Impairment Project [10]	4532	Australian	40–98	17.0	<−0.5D	2.1	<−5.0D	37.0	> 0.5D

*NR* not reported

**Fig. 4:**
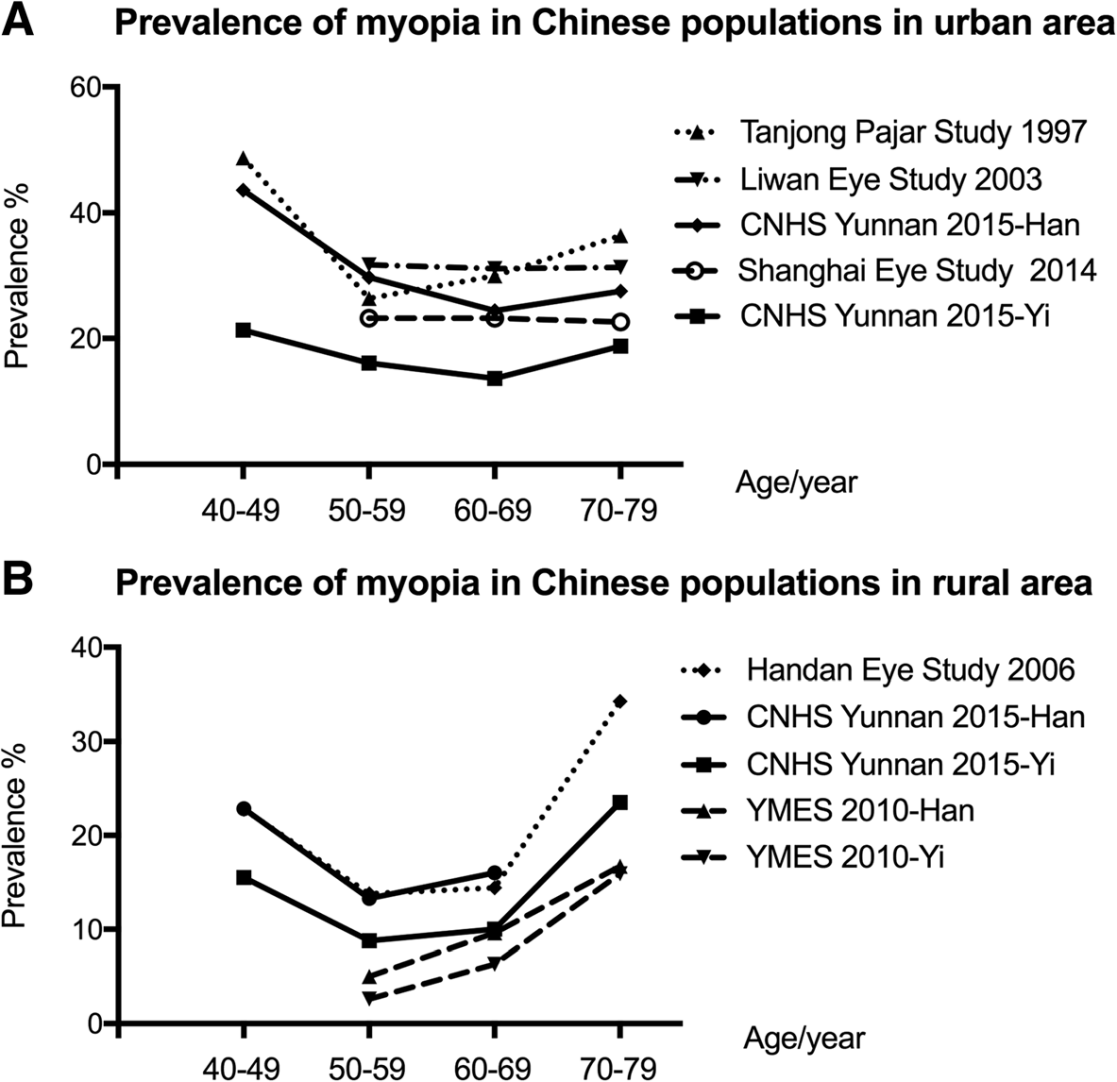
Prevalence of myopia in Chinese populations in selected studies. **a** Prevalence of myopia in Chinese populations in urban area; **b** Prevalence of myopia in Chinese populations in rural area. CNHS: Chinese National Health Survey; YMES: Yunnan Minorities Eye Study; Data for the age group of 70–79 years in the Han CNHS Yunnan 2015 study are not shown because of the limited number of samples (*n* < 15).

The Yunnan Minority Eye Study conducted in 2010, which focused only on myopia in those older than 50 years in rural areas, found a much lower prevalence of myopia than those in other studies of Chinese people [17]. However, it is difficult to draw a conclusion while comparing data under rural area settings to data from urban areas or urban/rural areas. Our study revealed that the age-standardized prevalence of myopia in the Han population was nearly double that in the Yi population (31.50% vs 16.80%, *p* < 0.0001). The Han population constitutes 91.6% of the population in China, and this proportion is even higher in Beijing and Southeast China [20]. Han is also the main ethnicity of Singaporean Chinese. To make the prevalence of myopia more comparable, we further calculated the prevalence of myopia separately under rural or urban settings for different age ranges (Fig. 4). We found that in urban settings, the prevalence of myopia in the Han population in our study was similar to those of the Tanjong Pajar study in Singapore, [5] slightly lower than that of the Liwan study in Guangzhou [14] and slightly higher than that in the Shanghai Eye Study (Fig. 4a) [12]. Similarly, under rural settings, the prevalence of myopia in the Han population in our study was nearly the same as that in the Handan Eye Study (Fig. 4b) [16]. Therefore, the Han population in underdeveloped Southwest China has a similar prevalence of myopia to those in East China or Singapore under urban or rural settings. If we only focused on subjects older than 50 years old in rural areas, the age-standard myopia prevalence was 14.3% in the Han population (95%CI 9.2–19.4%) and 11.5% (95%CI 7.0–16.0%) in the Yi population, which is higher than the results (Han 10.3, 95%CI 9.0–11.7%; Yi 8.1, 95%CI 6.8–9.4%) in the previous Yunnan Minority Eye Study under the same rural settings and age ranges [17]. This difference may have resulted from selection bias between different studies or from a possible increase in myopia prevalence over the last five years [25].

The age-standardized prevalence of hyperopia in our study was 19.89% (95%CI 18.16–21.61%) and was lower in the Han population than in the Yi population (16.58% vs 27.37%, *p* < 0.0001). With the multivariate logistic regression, compared to Yi ethnicity, Han ethnicity was found to be a protective factor in our study after being adjusted for age, sex, height, time spent in rural areas, education level, activity level, occupation, income, pterygium of the same eye, diabetes and smoking practice (*p* = 0.025). In a multiethnic study in Singapore, the Chinese population had the lowest hyperopia risk compared to that of Malay and Indian subjects [11]. Compared to studies focusing on populations older than 40 years and defining hyperopia as SE > 0.5D, our study found that the prevalence of hyperopia, especially in the Han population, was much lower than that in western countries, [5, 10, 26] Singapore, [27, 28] Japan, [6] Iran [29] and Mongolia (Table 4) [30].

Our study further identified several risk factors for myopia and hyperopia. For myopia, the prevalence of myopia first decreased and then increased with increasing age. A similar U-shaped curve was also reported in the Handan Eye Study, [16] Tanjong Pajar study, [5] Malay Eye Study in Singapore, [28] and Sumatra study in Indonesia [31]. The decreasing part of the U-curve could be explained by the intrinsic age-related decrease in the amount of an individual’s myopia [32] and/or cohort effect by which younger generations may have more reading exposure, less outdoor activity and other factors varying among birth cohorts [11]. Moreover, the increasing part of the U-curve might be related to the myopic shift in refraction due to cataract development [33]. Previous studies have indicated that compared to urban residence, rural residence could decrease the risk of myopia [13, 34, 35]. Considering the rapid urbanization process and that myopia development mainly happens in adolescence, we combined the residence information into time spent in rural areas, which was found to decrease the risk of myopia (*p* = 0.014) in our study. Education level was associated with a higher risk of myopia in our study, which has frequently been described in previous studies [36–38]. Moreover, we found that height was associated with myopia and that a taller height was protective. A relatively consistent view has been formed that height is positively related to the axial length of the eye [39–41]. However, the relationship between height and myopia is still controversial [9, 39, 42]. Our study also found that diabetes increases the risk of myopia significantly, as has previously been proven in the Los Angeles Latino Eye Study, [43] Barbados Eye Study, [26] and Handan Eye Study [16]. Furthermore, a higher level of HbA1c is a risk factor for myopia in patients with type 2 diabetes [44]. This relationship might be explained by the pathological change caused by the high level of blood glucose. Additionally, no relationship was found in some studies between diabetes and myopia [5, 45–47]. We found no association between myopia and activity level, occupation, and income level, which is consistent with the shift in the view that outdoor activities play a greater role than the young-age near-work model in decreasing the risk of myopia [48].

For hyperopia, the hyperopic shift with ageing has been proven in many longitudinal studies, [49–51] we found an increasing trend for the prevalence of hyperopia in both Han and Yi populations with an increasing age, and older age was found to be a significant risk factor for hyperopia. Additionally, the presence of a pterygium was found to increase the risk of hyperopia (*p* = 0.019), which was consistent with previous studies [52, 53]. A thinner cornea and sclera and more redundant conjunctivas were found in eyes with myopia compared to eyes with hyperopia, which might be protective from pterygium [54–56]. Pterygium is also related to older age, and the association between older age and hyperopia might also contribute [57].

The strength of this study included a relatively comprehensive population-based sample from a large city, county site and rural area; reasonable response rates; a standardized refraction assessment; and reliable demographic data. However, as this was a cross-sectional study, we were unable to establish causality between risk factors and RE. Cohort studies are recommended for the future. Moreover, further studies involving more participants in West China are highly recommended to draw an overall map of the understanding of RE in China.

## Conclusions

Our study investigated the overall prevalence of RE including myopia, hyperopia, high myopia and astigmatism in rural and urban areas in Yunnan China in adults aged 40–80 years. The Han population had a higher prevalence of myopia, high myopia and astigmatism but a lower risk of hyperopia than the Yi population. With multivariate logistic regression, myopia was found to be associated with age, height, time spent in rural areas, education level, and diabetes. Hyperopia was associated with ethnicity, age, time spent in rural areas and pterygium. The Han population in underdeveloped Southwest China in our study had a similar prevalence of myopia to those in East China or in Chinese Singaporeans under urban or rural settings.

## Abbreviations

BIA: Bioelectrical impedance analysis
BMI: Body mass index.
CNHS: China National Health Survey.
RE: Refractive error.
SAS: Statistical Analysis System.
SE: Spherical equivalent.
